# Supplementary material for: Macrophage innate training induced by IL-4 and IL-13 activation enhances OXPHOS driven anti-mycobacterial responses
Source: eLife. 2022 Sep 29;11:e74690. doi: 10.7554/eLife.74690 (PMC9555863; doi:10.7554/eLife.74690)

Image ID: 0003286_01
Acquire Time: 15-May-2022 17:40:07

Acquisition Information

#	Image ID	Acquire Time	Channels	Integration Times	Analysis	Image Name	Comment	Image Modifications
1	0003286_01	15-May-2022 17:40:07	700 800	10:00 10:00	Manual	0003286_01		

Image Display Values

Channel	Color	Minimum	Maximum	K
700	Gray Scale (Black on White)	0.000264	0.0571	0

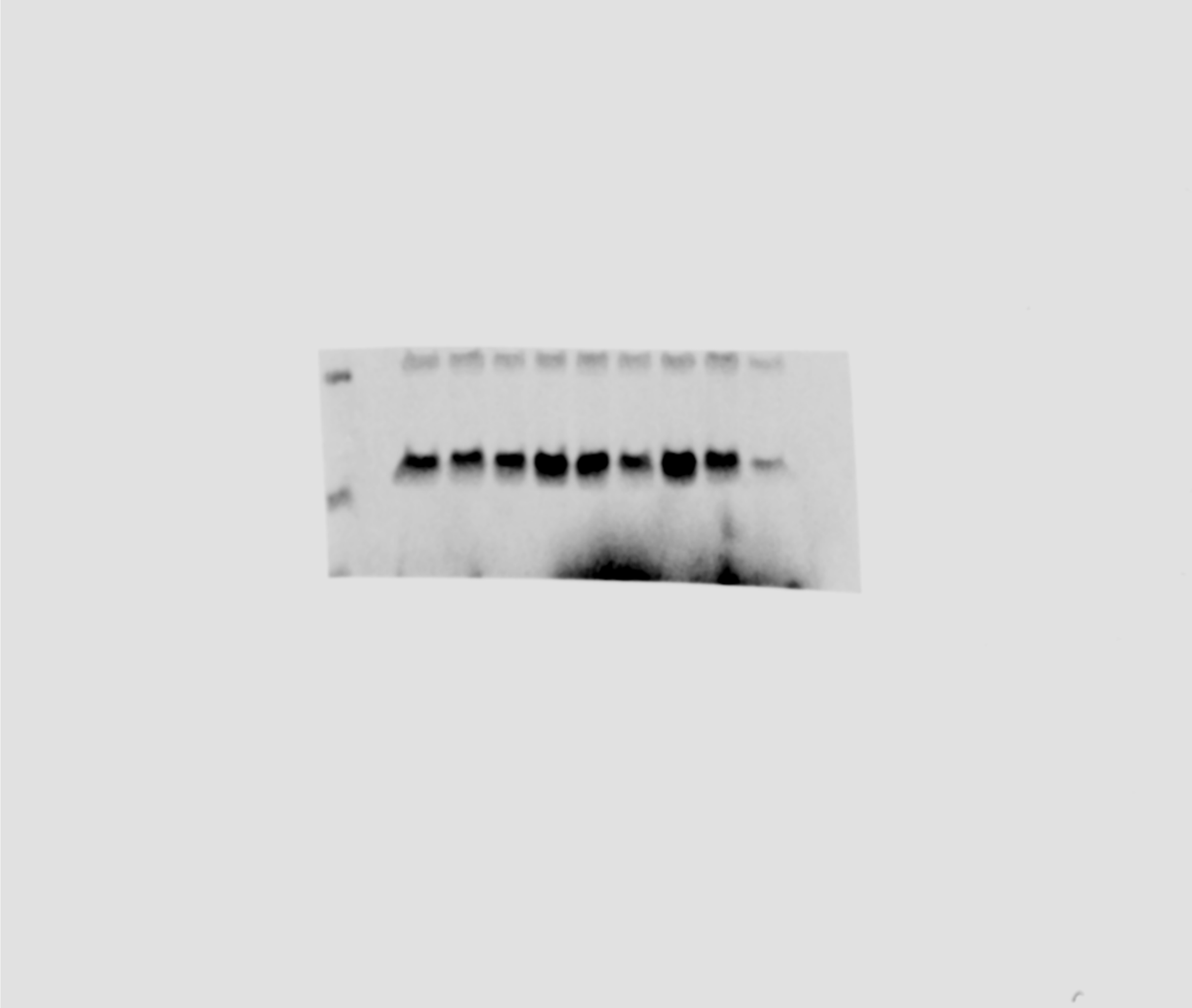

Supplement: Figure 2—figure supplement 3—source code 1. — Unedited western blots for histone H3, H3K4me3, H3K27me3 and H3K9me2, accompanying ponceau stains and figure where size and wells are labelled. [file elife-74690-fig2-figsupp3-code1.zip › Figure 2 figure supplement 3 source data 1/H3 blot (1).pdf]
