# Supplementary figures and images for: Macrophage innate training induced by IL-4 and IL-13 activation enhances OXPHOS driven anti-mycobacterial responses
Source: eLife. 2022 Sep 29;11:e74690. doi: 10.7554/eLife.74690 (PMC9555863; doi:10.7554/eLife.74690)

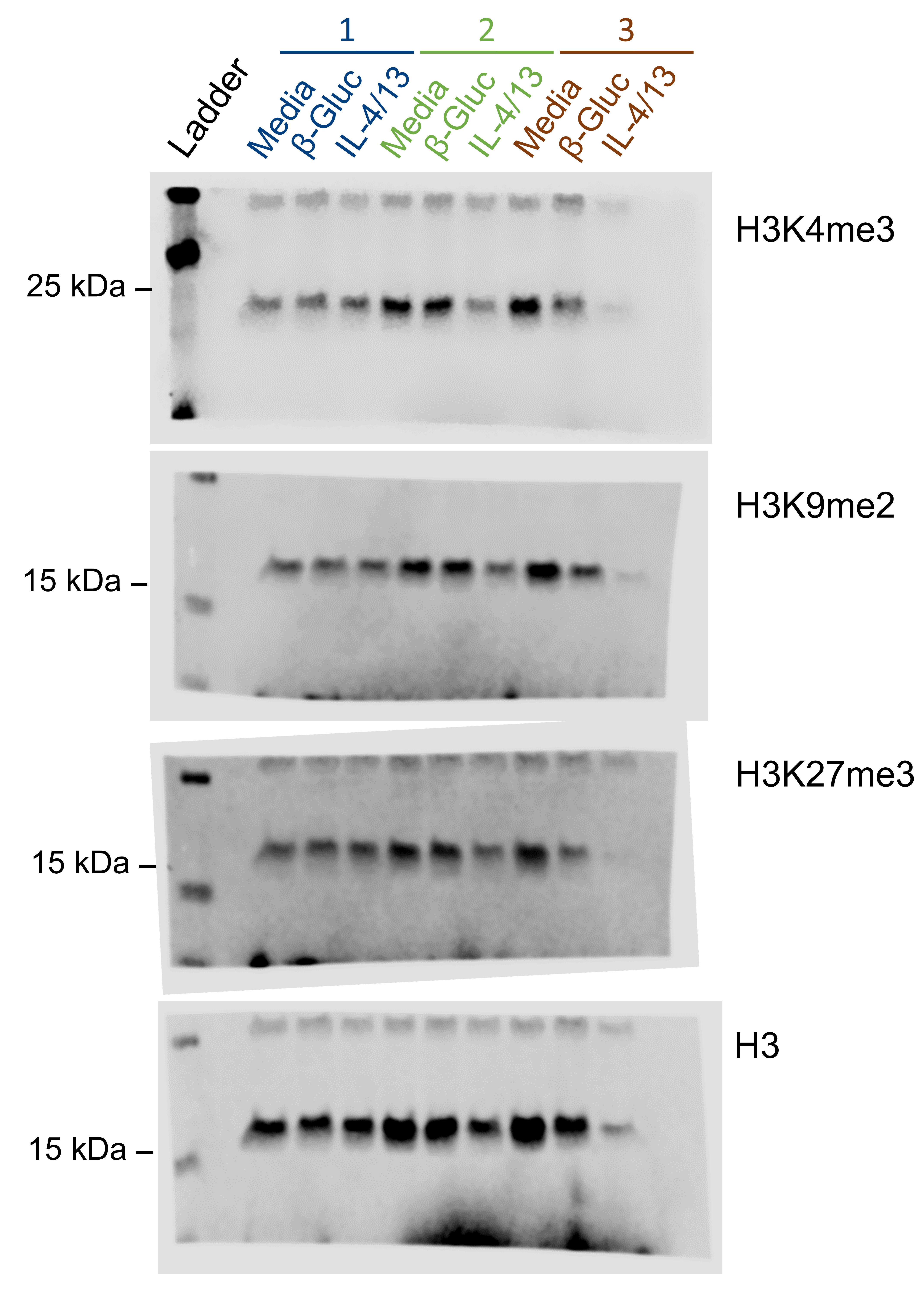

Supplement: Figure 2—figure supplement 3—source code 1. — Unedited western blots for histone H3, H3K4me3, H3K27me3 and H3K9me2, accompanying ponceau stains and figure where size and wells are labelled. [file elife-74690-fig2-figsupp3-code1.zip › Figure 2 figure supplement 3 source data 1/Fig 2 figure supplement 3 source data.tif]

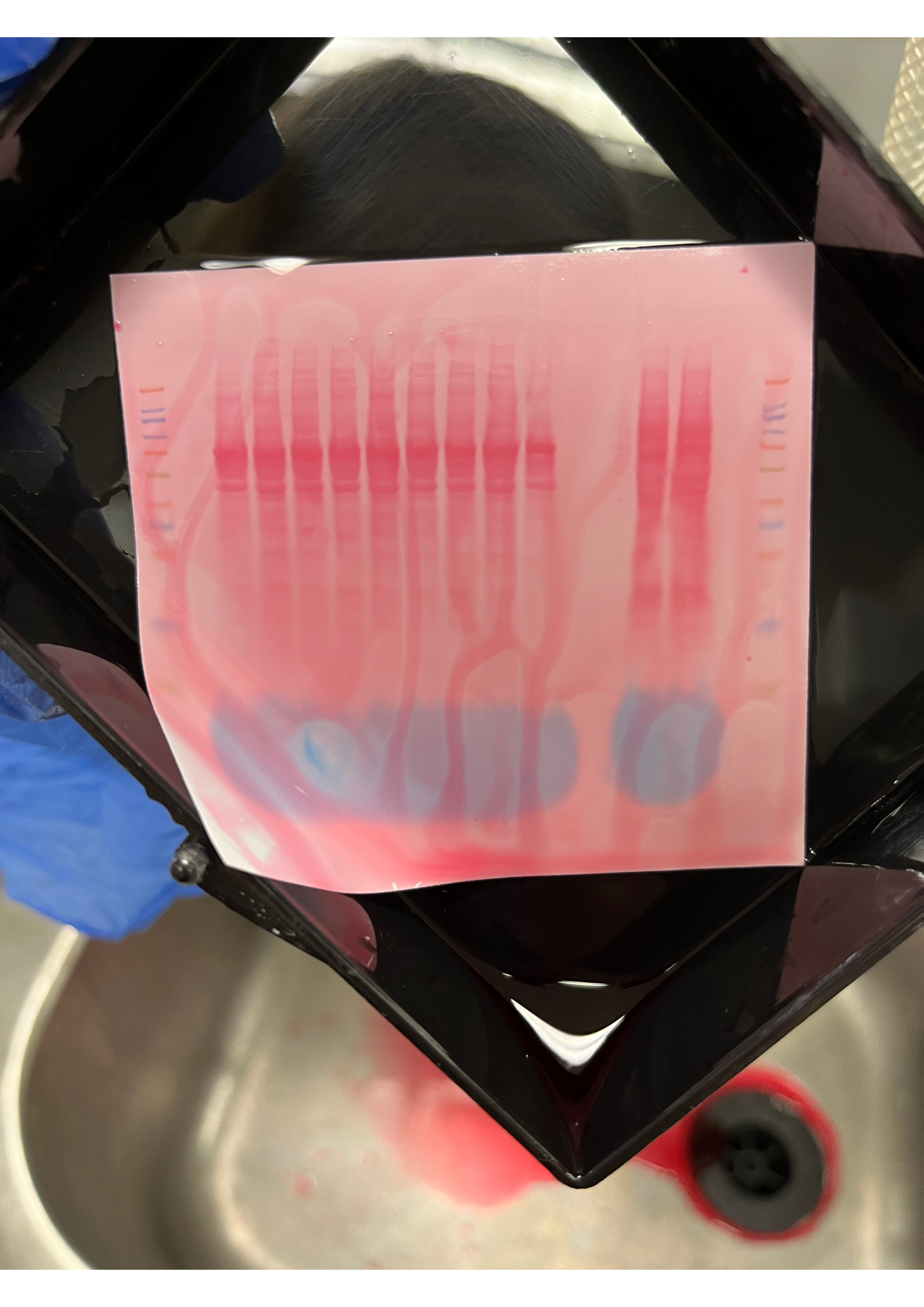

Supplement: Figure 2—figure supplement 3—source code 1. — Unedited western blots for histone H3, H3K4me3, H3K27me3 and H3K9me2, accompanying ponceau stains and figure where size and wells are labelled. [file elife-74690-fig2-figsupp3-code1.zip › Figure 2 figure supplement 3 source data 1/H3K27me3 Ponceau (1).pdf]

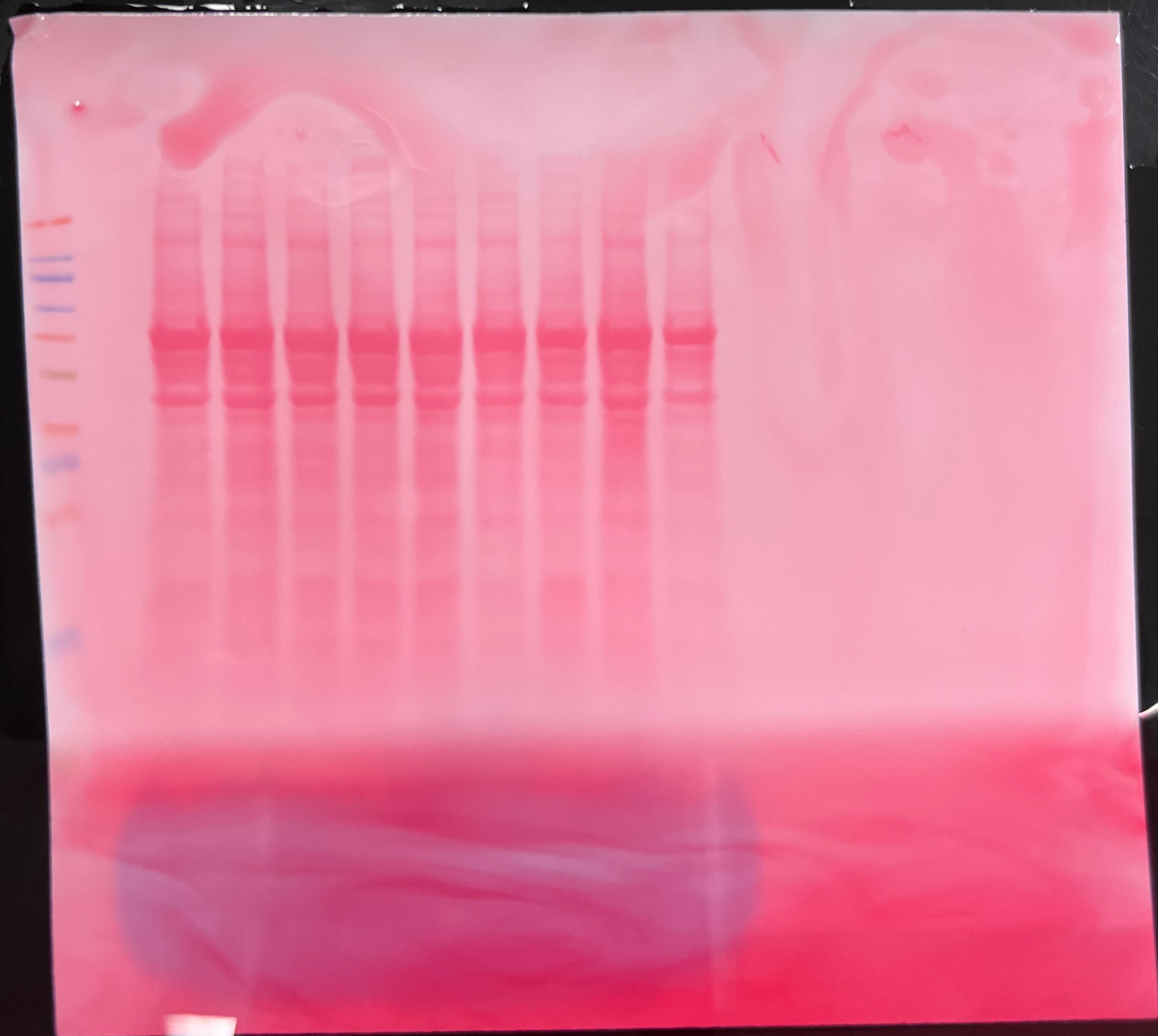

Supplement: Figure 2—figure supplement 3—source code 1. — Unedited western blots for histone H3, H3K4me3, H3K27me3 and H3K9me2, accompanying ponceau stains and figure where size and wells are labelled. [file elife-74690-fig2-figsupp3-code1.zip › Figure 2 figure supplement 3 source data 1/H3K4me3 & Total H3 Ponceau (1).pdf]

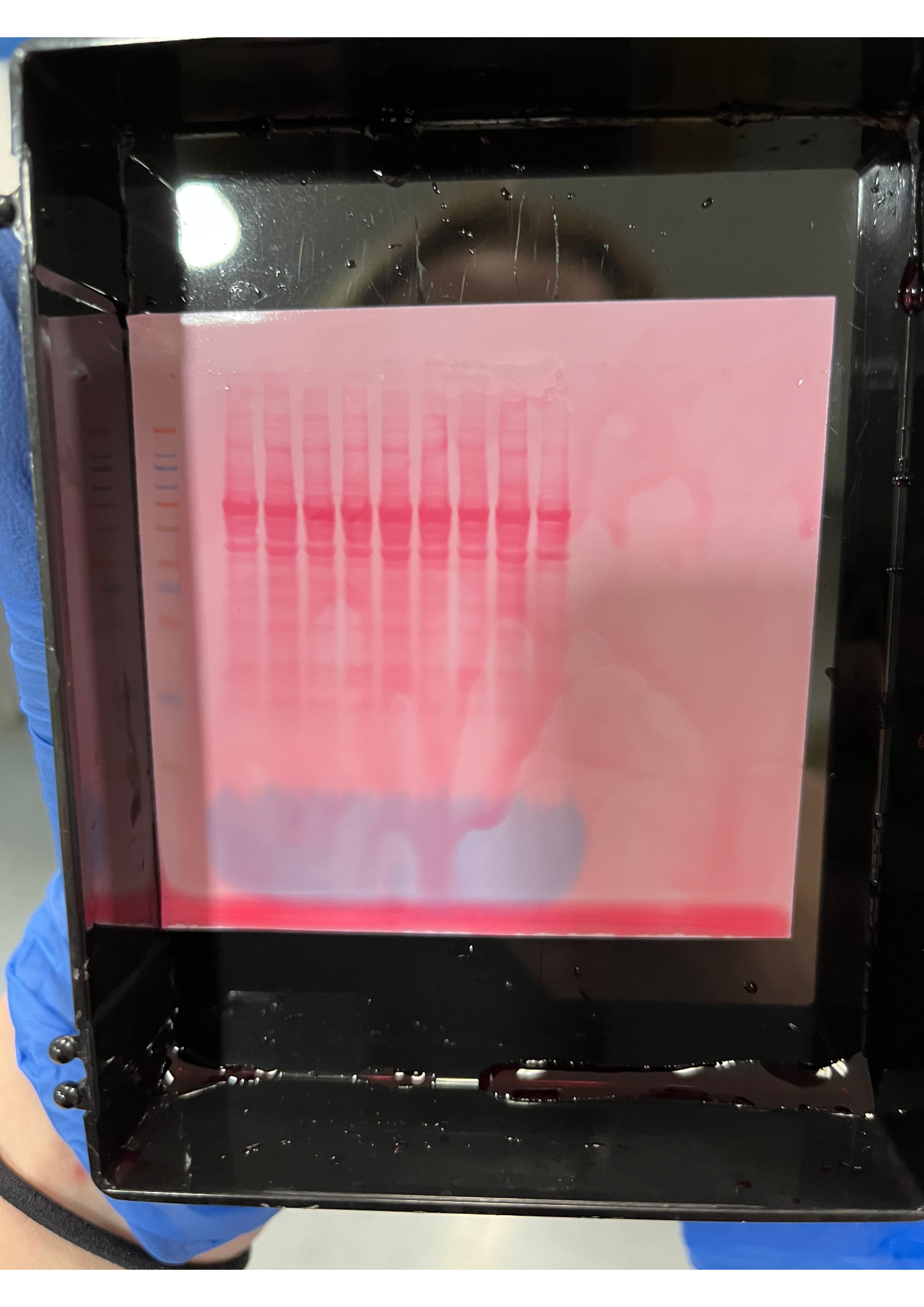

Supplement: Figure 2—figure supplement 3—source code 1. — Unedited western blots for histone H3, H3K4me3, H3K27me3 and H3K9me2, accompanying ponceau stains and figure where size and wells are labelled. [file elife-74690-fig2-figsupp3-code1.zip › Figure 2 figure supplement 3 source data 1/H3K9me2 Ponceau (1).pdf]
