# Supplementary material for: Macrophage innate training induced by IL-4 and IL-13 activation enhances OXPHOS driven anti-mycobacterial responses
Source: eLife. 2022 Sep 29;11:e74690. doi: 10.7554/eLife.74690 (PMC9555863; doi:10.7554/eLife.74690)
Supplement: Transparent reporting form [file elife-74690-transrepform1.docx]

***eLife’s* transparent reporting form**

We encourage authors to provide detailed information *within their submission* to facilitate the interpretation and replication of experiments. Authors can upload supporting documentation to indicate the use of appropriate reporting guidelines for health-related research (see [EQUATOR Network](http://www.equator-network.org/%20)), life science research (see the [BioSharing Information Resource](https://biosharing.org/)), or the [ARRIVE guidelines](http://www.plosbiology.org/article/info:doi/10.1371/journal.pbio.1000412) for reporting work involving animal research. Where applicable, authors should refer to any relevant reporting standards documents in this form.

If you have any questions, please consult our Journal Policies and/or contact us: [editorial@elifesciences.org](mailto:editorial@elifesciences.org).

**Sample-size estimation**

- You should state whether an appropriate sample size was computed when the study was being designed
- You should state the statistical method of sample size computation and any required assumptions
- If no explicit power analysis was used, you should describe how you decided what sample (replicate) size (number) to use

Please outline where this information can be found within the submission (e.g., sections or figure legends), or explain why this information doesn’t apply to your submission:

For tissue culture based-experiments the norm is to have 3 biological replicates and this standard was applied to this work. Given that this is the norm for this type of experiment, this was not directly stated nor was any power calculation carried out. The only exception are the BCG infection studies, where it is highlighted that what is presented are representative experiments, and this was done due to the variability in MOI counts between experimental replicates.

**Replicates**

- You should report how often each experiment was performed
- You should include a definition of biological versus technical replication
- The data obtained should be provided and sufficient information should be provided to indicate the number of independent biological and/or technical replicates
- If you encountered any outliers, you should describe how these were handled
- Criteria for exclusion/inclusion of data should be clearly stated
- High-throughput sequence data should be uploaded before submission, with a private link for reviewers provided (these are available from both GEO and ArrayExpress)

Please outline where this information can be found within the submission (e.g., sections or figure legends), or explain why this information doesn’t apply to your submission:

Biological/experimental replicates are labelled as “n” in figure legends and in the accompanying dataset, it is outlined how many biological and technical replicates there were per experiment/graph. Biological replicates are presented in rows (labelled “repeats”), and technical replicates are in the relevant columns.

No outliers were excluded, but negative values were either excluded or converted to 0, and these cases are outlined in the accompanying dataset.

**Statistical reporting**

- Statistical analysis methods should be described and justified
- Raw data should be presented in figures whenever informative to do so (typically when N per group is less than 10)
- For each experiment, you should identify the statistical tests used, exact values of N, definitions of center, methods of multiple test correction, and dispersion and precision measures (e.g., mean, median, SD, SEM, confidence intervals; and, for the major substantive results, a measure of effect size (e.g., Pearson's r, Cohen's d)
- Report exact p-values wherever possible alongside the summary statistics and 95% confidence intervals. These should be reported for all key questions and not only when the p-value is less than 0.05.

Please outline where this information can be found within the submission (e.g., sections or figure legends), or explain why this information doesn’t apply to your submission:

The norm for comparing sets of data is to use a student’s t-test, so this test was used throughout this work. The only exception was with BCG infection studies where multiple t-tests were used to account for the different time points. As the choice of test is a standard, it was not directly justified.

Raw data is presented in figures and the accompanying dataset.

Each figure legend states which statistical test was used, the number of biological/experimental repeats (n) and whether the data is representing mean +/- SD or SEM.

Exact p-values of all values ≤0.5 can be found in the accompanying dataset.

(For large datasets, or papers with a very large number of statistical tests, you may upload a single table file with tests, Ns, etc., with reference to sections in the manuscript.)

**Group allocation**

- Indicate how samples were allocated into experimental groups (in the case of clinical studies, please specify allocation to treatment method); if randomization was used, please also state if restricted randomization was applied
- Indicate if masking was used during group allocation, data collection and/or data analysis

Please outline where this information can be found within the submission (e.g., sections or figure legends), or explain why this information doesn’t apply to your submission:

This information does not apply for this study, as the same primary cells were given all treatment conditions of interest.

**Additional data files (“source data”)**

- We encourage you to upload relevant additional data files, such as numerical data that are represented as a graph in a figure, or as a summary table
- Where provided, these should be in the most useful format, and they can be uploaded as “Source data” files linked to a main figure or table
- Include model definition files including the full list of parameters used
- Include code used for data analysis (e.g., R, MatLab)
- Avoid stating that data files are “available upon request”

Please indicate the figures or tables for which source data files have been provided:

Metaboanalyst was used to analyse the metabolomics data; as metaboanalyst uses R as the statistical analysis tool, the R-history is included in the accompanying dataset.
